# Alteration of Iron Concentration in Alzheimer’s Disease as a Possible Diagnostic Biomarker Unveiling Ferroptosis

**DOI:** 10.3390/ijms22094479

**Published:** 2021-04-25

**Authors:** Eleonora Ficiarà, Zunaira Munir, Silvia Boschi, Maria Eugenia Caligiuri, Caterina Guiot

**Affiliations:** 1 Department of Neurosciences, University of Turin, 10124 Turin, Italy; zunaira.munir@unito.it (Z.M.); silvia.boschi@unito.it (S.B.); caterina.guiot@unito.it (C.G.); 2 Neuroscience Research Center, University “Magna Graecia” of Catanzaro, 88100 Catanzaro, Italy; me.caligiuri@unicz.it

**Keywords:** iron, biomarkers, ferroptosis, neurodegeneration, Alzheimer’s disease

## Abstract

Proper functioning of all organs, including the brain, requires iron. It is present in different forms in biological fluids, and alterations in its distribution can induce oxidative stress and neurodegeneration. However, the clinical parameters normally used for monitoring iron concentration in biological fluids (i.e., serum and cerebrospinal fluid) can hardly detect the quantity of circulating iron, while indirect measurements, e.g., magnetic resonance imaging, require further validation. This review summarizes the mechanisms involved in brain iron metabolism, homeostasis, and iron imbalance caused by alterations detectable by standard and non-standard indicators of iron status. These indicators for iron transport, storage, and metabolism can help to understand which biomarkers can better detect iron imbalances responsible for neurodegenerative diseases.

## 1. Introduction

Iron is a *d*-block transition metal with reactive properties and with excellent redox potential. It can readily donate and accept electrons to participate in oxidation-reduction reactions that are essential for a number of fundamental biological processes [1]. Iron exists in two ionic states, Fe^3+^ and Fe^2+^. Free (unbound) iron can be toxic since it readily combines with oxygen and nitric oxide, catalyzing the formation of a highly reactive hydroxyl group (OH-) and peroxynitrite (ONOO-), resulting in oxidative and nitrosative damage to proteins, lipids, and nucleic acids [2].

Therefore, most of the circulating and the stored iron is linked to proteins and other transporters, and cells are equipped with proteins for iron uptake to secure its vital functions and limit its potential toxicity. Not only does iron binding to glycoprotein ligands prevent toxicity, but the nature of the ligands finely modulates the redox potential of iron.

The crucial role of iron in health and diseases is long recognized together with its very sensitive distribution in the human body and sophisticated pathways to import, chaperone, sequester, and export iron in order to maintain an appropriate balance [3]. 

Healthy adult bodies contain 4–5 g of iron. Iron is mostly (65%) bound in red blood cell hemoglobin (Hb), and 30–35% is stored in the liver in the form of ferritin. Iron is also in the form of iron–sulfur clusters or heme in enzymes and multiprotein complexes [4,5]. The body absorbs 1–2 mg of dietary iron a day, and this intake is balanced with losses in the form of sloughed intestinal mucosal cells and other blood losses [6].

Iron is an essential micronutrient due to its relevance in the process of erythropoiesis, oxidative metabolism, and cellular immune responses [7]. In humans, iron is incorporated into proteins as a component of heme (e.g., hemoglobin, myoglobin, cytochrome proteins, myeloperoxidase, nitric oxide synthetases), iron–sulfur clusters (e.g., respiratory complexes I–III, coenzyme Q_10_, mitochondrial aconitase, DNA primase), or other functional groups [8]. These iron-containing proteins are required for vital cellular and organism functions, including oxygen transport, mitochondrial respiration, intermediary and xenobiotic metabolism, nucleic acid replication and repair, host defense, and cell signaling. The remaining iron-dependent proteins are specifically involved in iron absorption (divalent metal transporter-1, DMT1)), export (ferroportin, Fpn), storage (ferritin, Ft), and transport (transferrin, Tf).

The complex and multi-hierarchy iron metabolism may be divided into different steps:

(a) Active transport of dietary iron in the gastrointestinal tract by enterocytes for iron entry into the bloodstream;
(b) Transport of iron through the bloodstream;
(c) Entry of iron into different tissues and cells;
(d) Regulation of intracellular levels, trafficking, and metabolization of iron.

**Figure 1:**
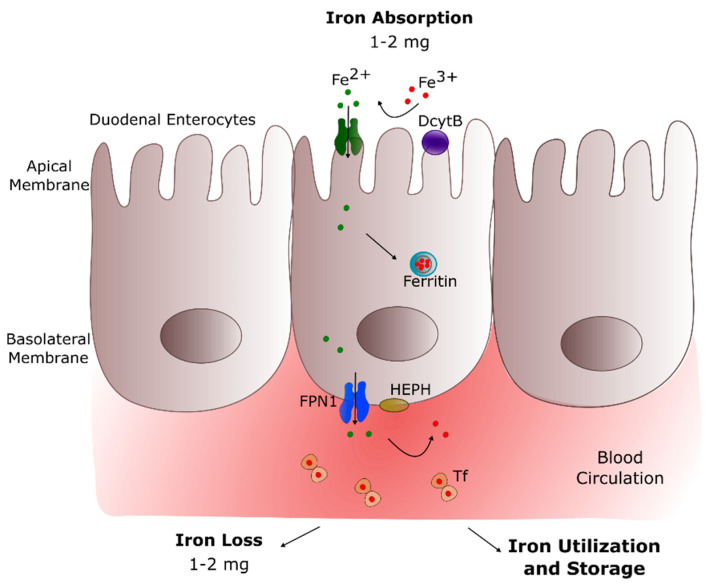
Mechanism of systemic iron metabolism. After being reduced by DcytB at the apical membrane of duodenal enterocytes, dietary iron is absorbed by DMT1 and driven to the basolateral membrane of these cells; iron is exported by FPN1 to the circulation, transformed from ferrous to ferric iron by HEPH, and finally transported by Tf in the blood. Abbreviations, DcytB—duodenal cytochrome B, DMT1—divalent metal transporter-1, FPN1—ferroportin-1, HEPH—hephaestin, Tf—transferrin.

The major route of iron acquisition is intestinal absorption (Figure 1), where dietary Fe^3+^ is reduced to Fe^2+^ by the ferrireductase enzyme duodenal cytochrome B (DcytB), localized at the apical surface of enterocytes [9]. The divalent Fe^2+^ ions enter the duodenal enterocytes via the divalent metal transporter 1 (DMT1), a duodenal brush-border membrane protein specific for ferrous iron, zinc(II), and copper(II) [10]. This transport is proton(H^+^)-coupled and depends on the presence of luminal H^+^ ions. When there is a low demand for iron in the body, iron is stored within the enterocytes in the form of ferritin, an intracellular iron storage protein [11].

Ferritin is a ubiquitous, mainly cytosolic, globular protein of 450 kDa comprising 24 subunits of Ft-H and Ft-L chains [12,13]. Ft-H possesses an active ferroxidase center that catalyzes the oxidation of Fe^2+^ to the Fe^3+^ form, while Ft-L promotes its nucleation within the protein shell for storage. Together, these chains form a nano-cage storing approximately 4500 Fe^3+^ ions in a bioavailable and non-toxic form (as mineral ferrihydrite) [14,15,16]. The precise mechanism of binding, storage, and release of iron from ferritin requires further clarification. Available information suggests that a cytosolic iron chaperone Poly (rC)-binding protein 1 binds cytosolic iron for delivery to ferritin [17]. Ferritin-binding proteins, amino acids, and small molecules regulate the release of iron from ferritin, supporting the gated pore model but requiring further characterization [18,19,20]. Stored iron is released in response to low intracellular iron, thus maintaining equilibrium between ferritin iron and free iron, which constitutes the so-called labile iron pool (LIP).

On the other hand, when iron demand is high, the absorbed ferrous iron is transported across the basolateral membrane into blood. This phase is controlled by ferroportin 1 (FPN1), a ferrous iron export protein modulating the quantity of enterocyte iron absorbed into the circulation and available to the body [21].

Iron transportation in the bloodstream is performed by the plasma protein transferrin (Tf) [22], which requires the transformation of ferrous iron back to ferric iron. This step is achieved by hephaestin (HEPH), a multi-copper ferroxidase enzyme anchored to the basolateral enterocyte membrane and coupled to FPN1, which catalyzes the oxidation of Fe^2+^ to Fe^3+^ ions [23]. Then, Fe^3+^ binds to Tf, which has a high affinity for Fe^3+^ and enables iron transport around the body organs, e.g., in the brain (Table 1). Apotransferrin (apo-Tf) is the unbound form of this transporter and contains two ferric binding sites, of which none, one, or both may be filled. Under physiological conditions, only about 30% of Tf is saturated [1]. Tf is the primary iron-transport protein with a half-life of 8 to 10 days that reflects both protein and iron status. Iron with Tf maintains Fe^3+^ in a soluble form under physiological conditions, facilitates regulated iron transport and cellular uptake, and maintains Fe^3+^ in a redox-inert state, preventing the production of toxic free radicals [24].

## 2. Iron in Brain

Brain iron levels are tightly regulated to ensure the normal function of the central nervous system (CNS) and to prevent high sensitivity of CNS to toxicity [25,26].

**Figure 2:**
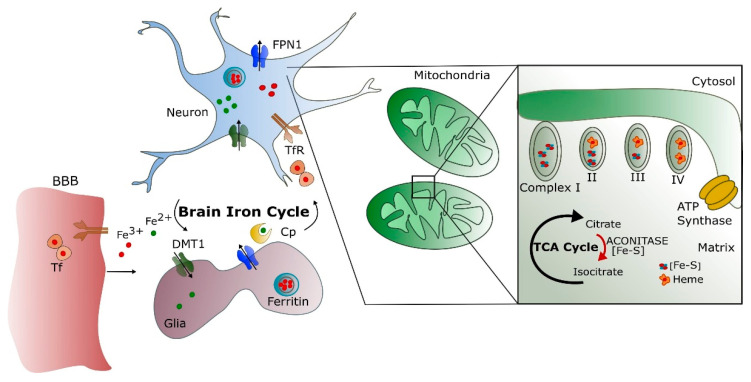
Summary of brain iron cycle and mitochondrial functions. Iron crosses the BBB via the TfR pathway on the endothelium. The brain iron cycle involves glia and neurons. Astrocytes extend long processes that enclose the brain capillaries and help to form the BBB. The DMT1 transports iron. Near the ends of these processes, a special form of the Fe oxidizing enzyme, Cp, is expressed. Iron binds to Tf circulating in CSF and enters the neurons mainly by TfR. The cells export iron mainly by FPN1. Into the cells, the mitochondrial electron transport chain contains multiple iron–sulfur (Fe–S) clusters and heme-containing proteins necessary for ATP synthesis. NADH dehydrogenase (complex I) contains Fe–S clusters, succinate dehydrogenase (complex II) contains Fe–S clusters and one heme moiety, while complex III (cytochrome bc1) contains Fe–S cluster and several heme groups vital for its functions. Complex IV (cytochrome c oxidase) also contains two heme moieties. Aconitase is a key enzyme, catalyzing the reaction of citrate to isocitrate in the tricarboxylic acid (TCA) cycle and containing Fe–S clusters.

The brain is among the most metabolically active organs in the body and accounts for at least 20% of the body’s energy consumption, although it represents only about 2% of its weight. Iron plays a fundamental role during ATP production as a cofactor in the oxidative chain for cytochromes and iron–sulfur complexes [27]. About 75–80% of the energy supports neuronal activity, with the remainder utilized to sustain the functions of astrocytes, oligodendrocytes, and microglia [28]. Both axonal and synaptic signaling need neuronal energy, but the major part is used post-synaptically [29]. The mitochondrial function must supply ATP, and iron is necessary to support oxidative phosphorylation (Figure 2).

Accordingly, an adequate supply of iron is necessary to sustain its high-energy needs [28,30,31]. Therefore, iron is the most abundant metal in the brain [32]. It is a co-factor involved in oxygen transportation, DNA synthesis, mitochondrial respiration, myelin synthesis, neurotransmitter synthesis, and metabolism [33,34] but can become neurotoxic when there is excessive intracellular accumulation [35,36]. The systemic organs and the brain share the same iron regulatory mechanisms and pathways based on iron-modulating proteins, providing a link to the maintenance of iron homeostasis within the brain [37].

The delicate balance of iron in the brain milieu is mainly maintained by the brain barrier systems, coordinating the direction of iron fluxes between the blood and the brain/cerebrospinal fluid (CSF) [38].

Brain iron concentrations are not static; they increase with age and in many diseases and decrease when iron is deficient in the diet. In vivo magnetic resonance imaging (MRI) shows that iron deposition increases in numerous age-dependent neurodegenerative diseases, such as Parkinson’s and Alzheimer’s disease (AD), and accumulates mainly in the basal ganglia [39,40]. Moreover, increased levels of iron are associated with motor and cognitive impairment in the elderly [41]. Iron is believed to enter the brain via the blood–brain barrier (BBB) by Tf receptor-mediated endocytosis in the brain capillaries and is released back to circulation via CSF [42]. Iron is present in various cell types in the CNS but is abundant in the astrocytes (star-shaped glial cells), which gave rise to the idea that glial cells are involved in iron storage and regulation [33].

### Brain Iron Metabolism

Blood iron entrance into the brain is controlled by the BBB [43] and, to a lesser extent, by the blood–cerebrospinal fluid barrier (BCSFB) [44]. The role of the BBB is to protect the brain from neurotoxic plasma components and pathogens [45] as well as to control the chemical composition of the neuronal milieu by regulating the transport of molecules required for normal neuronal functioning [46]. The BBB is formed by a monolayer of tightly sealed brain microvascular endothelial cells (BMVECs) extending along the vascular tree [47] and expressing low paracellular and transcellular permeability [48]. Those endothelial cells are surrounded by a basal lamina and astrocytic perivascular end-feet, forming the neurovascular unit [49]. Iron enters the BMVECs as a low molecular weight complex, or via transferrin receptor-1 (TfR1) mediated endocytosis of Tf, or independently as non-transferrin-bound iron (NTBI) in a multi-step transcellular pathway. The binding of Tf to Tf receptors (TfR) at the lumen of the brain microvasculature facilitates iron uptake via receptor-mediated endocytosis [26,50,51] (Figure 2).

The Tf/TfR1 pathway is considered to be the major route for iron transport across the luminal membrane of the capillary endothelium [50,52]. The complex passes through the cell in the endocytosis vesicle, where the acid environment facilitates the release of ferric iron from Tf and its reduction to ferrous iron by endosomal reductase [53], possibly DcytB. The next steps in this pathway are still not completely clear. One possibility is that ferrous iron is transported from the endosome to the cytosol by the DMT1 [54] and joins the intracellular labile iron pool (LIP) [55]. It could be further utilized for metabolic purposes by the endothelial cells, stored in endothelial cell ferritin [56], or imported into mitochondria via mitoferrins and TfR2 [57]. It could be also released into the extracellular fluid by the action of export protein ferroportin (Fpn) [58] and re-oxidized to Fe^3+^ by ferroxidases HEPH and ceruloplasmin (Cp) [43], also expressed on the end-foot processes [54]. Studies confirmed that the capillary endothelium of the BBB, the neurons, and the astrocytes has the ability to express Fpn and HEPH [59,60]. The alternative mechanism that is proposed is that the endosome containing the Tf–TfR1 complex reaches the abluminal side and releases iron between the endothelial cells and the astrocyte end-foot processes [26]. Oxidized iron binds to apo-Tf circulating within the brain [55]. The main source of Tf in the brain interstitium is its diffusion from ventricles and oligodendrocytes synthesize a certain amount [61]. Because of the low concentrations of Tf in the CSF, iron saturation of CSF Tf is almost 100%, while serum Tf is saturated by about 30% [26].

Different cell types in the brain acquire iron by distinct pathways. Neurons express high levels of TfR1. Therefore, Tf is the main source of iron for neurons [54], although neurons can also uptake NTBI from interstitial fluid. Oligodendrocytes and astrocytes express less TfR1, and NTBI can be a source of iron [53,62].

Due to their peculiar position in the BBB, astrocytes take up iron from the circulation and distribute it to other cells in the CNS using different pathways: by DMT1 to glial cells, by binding ferrous iron in the brain interstitium to ATP or citrate released from astrocytes and transported to oligodendrocytes and astrocytes as NTBI [26], and finally iron can be stored as ferritin in astrocytes and exported by a mechanism involving Fpn and Cp [63]. Since most of the accumulated iron is within astrocytes, mainly iron-loaded astrocytes can cause brain toxicity [64]. Oligodendrocytes, which are responsible for myelin production, need high amounts of ATP [65]. Many of the enzymes involved in ATP production require a supply of iron, such as pathways for cholesterol and fatty acid synthesis for the myelination, which are iron-dependent. Some of these enzymes (e.g., NADH dehydrogenase and HMG-CoA reductase) are abundant in oligodendrocytes instead of other CNS cell types [65]. A suitable supply of iron during myelination is needed; in fact, a dietary iron restriction decreases the amount and the composition of myelin during gestation and early post-natal periods [66].

## 3. Brain Iron Mis-Metabolism

Abnormal iron content or the presence of free iron (LIP) may alter the brain metabolism by producing oxidative stresses, which could contribute to neurodegeneration due to the high susceptibility of the brain to oxidative damage. Moreover, functional mutation(s) in iron-modulating proteins disrupt iron homeostasis in systemic organs and the brain to a varying extent and, in some cases, result in specific human disorders [33,67,68]. The correlation between brain iron metabolism and neurotoxicity associated with neurodegenerative conditions such as AD remains to be fully elucidated [69], however, much evidence highlighted the involvement of iron in neurodegeneration.

Increased concentrations of total iron with aging might be caused by several factors that include increased BBB permeability, inflammation, redistribution of iron within the brain, and changes in iron homeostasis [70]. Aging processes might compromise the iron homeostatic system, leading to an excess of iron that is not efficiently chelated by storage proteins or other molecules [71]. Total iron concentrations increase with age in substantia nigra, putamen, globus pallidus, caudate nucleus, and cortices, but why this increase is selective for some areas of the brain is unclear [72]. Regional distribution of total iron in a healthy adult brain is heterogeneous; the highest iron concentrations were detected in the basal ganglia (putamen, globus pallidus, and caudate nucleus), whereas low concentrations were detected in cortical grey matter, white matter, midbrain, and cerebellum, and the lowest iron concentrations were in pons, locus coeruleus, and medulla [73]. Regional heterogeneity of brain iron and its change with age were both confirmed in vivo by MRI [74].

The aggregation of proteins involved in neurodegenerative disorders was shown in vitro to be triggered by elevated ferric iron concentrations [75]. Inclusion bodies containing damaged or aggregated proteins could cause endoplasmic reticulum stress, which is a common feature of several neurodegenerative diseases [76].

Defective homeostasis of the redox-active metals iron and copper probably contributes to the neuropathology of AD. High concentrations of zinc, copper, and iron are present in the insoluble amyloid plaques and the neurofibrillary tangles characteristic of AD. Focal accumulation of zinc, copper, and iron might deprive other brain tissues of these essential metals, leading to aberrant neuronal function [77]. Abnormal homoeostasis of zinc, copper, and iron metal ions is implicated in the misfolding process associated with the production of amyloid β (Aβ) from amyloid precursor protein (APP) and hyperphosphorylated tau (found in plaques and tangles) and contributing to neuronal oxidative stress [78]. Increases in iron in animal brains produce pronounced cognitive defects [79]. Iron deposits in presenilin/APP transgenic mice models of AD [80] and colocalizes with Aβ plaques [81], and increases in total brain iron coincide with early plaque formation [82].

Some studies suggest non-specific co-precipitation of iron and other metals with aggregated proteins, while others associate brain iron directly with disease pathogenesis [83,84,85]. In addition, dysfunction of ferritin or absence of Cp alter brain iron homeostasis and can induce neurotoxicity [37].

### 3.1. Oxidative Stress and Ferroptosis

Iron can promote radical formation from physiological or xenobiotic compounds, e.g., by catalyzing autoxidation, initiating lipid peroxidation, and reacting with hydrogen peroxide with consequent production of more highly reactive and toxic species by means of Fenton reaction [86]. Consequently, excess iron stores may increase pro-oxidant reactions and generation of free radicals, inducing cellular death in neurodegeneration. Furthermore, iron can be involved in inflammation processes, which play a key role in mediating cellular death and destruction via poorly liganded iron [87].

In 2012, Dixon first proposed the concept of ferroptosis., i.e., iron-dependent cell death characterized by the accumulation of lipid ROS which is morphologically, biochemically, and genetically distinguished from other forms of cell death including apoptosis, necrosis, autophagy, and pyroptosis [88,89,90].

Morphologically, cell ferroptosis is featured by decreased mitochondrial volume, increased bilayer membrane density, and reduction or disappearance of mitochondrial cristae [88,89,91], but the cell membrane is preserved, and the nucleus remains normal in size. Biochemically, intracellular glutathione (GSH) depletion and decreased activity of glutathione peroxidase 4 (GPX4) mean lipid peroxides cannot be metabolized by the GPX4-catalyzed reduction reaction, and Fe^2+^ oxidizes lipids in a Fenton-like manner, resulting in a large amount of ROS, promoting ferroptosis [91,92,93]. Genetically, ferroptosis is a biological process involved in iron homeostasis and lipid peroxidation metabolism regulated by genes involved in iron metabolism via the Tf/TfR1 complex, the iron–sulfur cluster assembly enzyme, and the ferritin [88].

**Figure 3:**
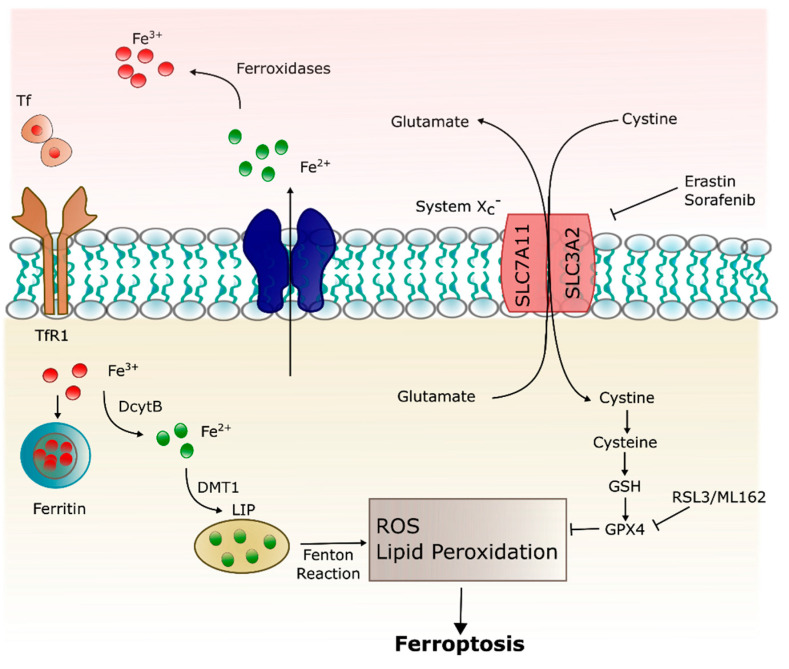
Ferroptosis pathway. Ferroptosis can be initiated through Tf endocytosis linked to TfR1. Ferric iron (also stored in ferritin) is released from the TfR1 complex and reduced to ferrous iron that can be stored in ferritin or remain into the cytoplasm as a labile iron pool (LIP). LIP is composed mainly by Fe^2+^, which can generate ROS through Fenton reaction and lipid peroxidation. Ferroptosis is inhibited by GPX4, which depends on GSH, synthesized via the entry of cystine into the cell by the system x_c_^−^.

Ferroptosis can be triggered by pharmacological impairment of anti-oxidant systems involving GSH and GPX4 [93]. The glutamate/cystine antiporter (x_c_^−^) exports cellular glutamate in exchange for extracellular cystine. Once inside the cell, cystine is converted to cysteine, a precursor of the endogenous antioxidant, GSH [88]. Erastin and sorafenib trigger ferroptosis via inhibition of x_c_^−^, depleting GSH and inactivating GPX4 [93,94]. Ferroptosis may also be induced by the administration of GPX4 inhibitors, RSL3 and ML162. GPX4 catalyzes potentially toxic lipid hydroperoxides to non-toxic lipid alcohols [95] and its inactivation via GSH depletion or direct GPX4 inhibition, inducing lipid peroxidation/oxidative stress and eventually cell death (Figure 3). Deferoxamine is able to prevent ferroptosis-induced cell death through quenching of excess iron [96,97].

The hallmarks of ferroptosis (iron dysregulation, lipid peroxidation, inflammation) are related to neurodegeneration and cognitive impairment [98,99].

### 3.2. Dysfunction of Ferritin

The physiological task of ferritin is to protect cells from the redox-active ferrous iron (Fe^2+^). Thus, under normal conditions, the response to a cytosol increase of Fe^2+^ is its rapid uptake into ferritin, where it is physiologically stored in a redox-inert form of iron oxyhydroxides containing only Fe^3+^ ions. The presence of ferrous iron inside pathological ferritin could reveal an alteration in the mineralization process, e.g., that the enzymatic oxidation process is faulty [100,101]. This process of Fe^2+^ oxidation inside ferritin takes place in specific ferroxidase sites in the Ft-H subunit [102]. Thus, dysfunction of ferritin could be a cause of the alterations in the mineralization of the ferritin cores, and this fact may be associated with aging processes and especially with neurological diseases, e.g., AD and Parkinson’s disease [103].

The imbalance of iron metabolism in affected regions also causes mitochondrial abnormalities, accumulating oxidatively damaged DNA in the mitochondria of a mouse model of hereditary ferritinopathy [104]. Interestingly, mutant Ft-L itself is targeted by ROS, resulting in its cleavage and disruption of the ferritin shell, confirming the role of free radicals in the process [105]. Co-aggregation of wild-type Ft chains can be initiated by the free radicals generated by mutant Ft-L, creating an iron imbalance in the brain [106].

Dysfunction of ferritin can be evaluated by measuring ferritin levels in biofluids, both in blood/serum and in CSF (in lower concentration than in the plasma). Several studies focused on these investigations in neurodegenerative diseases. Blood ferritin levels were reported significantly higher in patients with AD in comparison to healthy controls [107], and the incidence of serum ferritin levels above the normal range was significantly greater in individuals with AD [108]. Interestingly, different microscopy techniques were used to analyze the morphology of erythrocytes, showing relative substantial changes taken from high serum ferritin in AD individual and arguing that high ferritin levels may contribute to an accelerated pathology [109]. Serum/plasma ferritin was investigated as a preclinical marker of AD, reporting that both plasma and serum ferritin correlated positively with the neocortical amyloid-β load (NAL), underlining also a potential discriminating power between low and high NAL [110].

In addition, it was shown that ferritin levels in the CSF (reflecting brain status) can predict AD outcomes, being strongly associated with apolipoprotein E (APOE) levels and elevated by the AD risk allele APOE-ε4, thus revealing that elevated brain iron adversely impacts AD progression [111]. Moreover, ferritin levels are associated with longitudinal changes in CSF Aβ and Tau protein (the two hallmarks of AD), accelerating AD pathology [112].

### 3.3. Ceruloplasmin Deficiency

**Figure 4:**
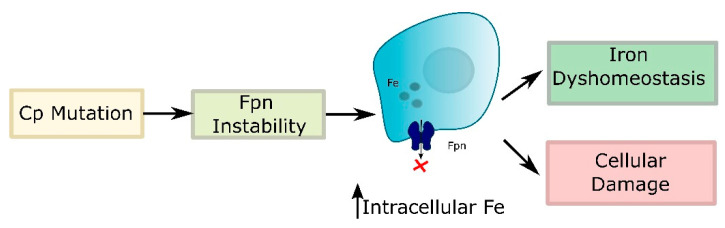
Absence or dysfunction of Cp destabilizes Fpn, resulting in intracellular accumulation of iron with consequent iron dyshomeostasis and cellular damage.

Cp is a copper-containing α2-glycoprotein that functions as a multi-copper ferroxidase to regulate body iron homeostasis [113]. The holoprotein contains six copper atoms that confer ferroxidase activity, which is responsible for the oxidation of Fe^2+^ released from intestinal epithelial, capillary endothelial, and reticuloendothelial cells to Fe^3+^, thereby modulating iron transport at multiple sites. Within the brain, Cp is expressed mainly on glial cells in the cerebellum, the microvasculature [114], and the inner nuclear layer of the retina [115]. Although Cp requires copper for its function, the absence of Cp causes an imbalance of iron metabolism rather than a disturbance of copper homeostasis [116]. The absence of Cp causes iron overload mainly by down-regulating Fpn, the iron export protein that is stabilized by the ferroxidase activity of Cp [117]. It is likely that ROS-mediated down-regulation of Cp and perhaps Fpn contribute to the neurotoxicity associated with neurodegenerative conditions (Figure 4). Currently, the neurotoxicity of iron accumulation resulting from the insufficiency or the decreased activity of Cp is considered as one of the mechanisms in the development of neurodegenerative diseases, such as AD and Parkinson’s disease [118].

### 3.4. Dysfunction of Transferrin

The importance of Tf in neurological diseases is underappreciated, though iron dyshomeostasis leading to iron overload is reported in a number of neurodegenerative diseases [119,120,121]. The cause of the age-related iron overload is still unknown but may be due to a change in iron delivered via Tf or an alteration of iron efflux mechanisms from the brain. Contributing to the lack of knowledge about the role of Tf in age-related neurodegenerative diseases is the incomplete understanding of the signals related to iron transport across the BBB. Tf secretion into the CSF may be altered in states of iron dyshomeostasis, and evaluation of Tf in CSF may be useful in determining the iron status of the brain. For example, there are elevated levels of Tf and reduced ferritin in the CSF of patients with restless legs syndrome (RLS), and this is indicative of low brain iron in patients with idiopathic RLS [122].

Brain iron can also be reduced in ID that frequently results in hypomyelination [123]. ID can result in cognitive and motor impairments that last throughout life. It is hypothesized that the deficits associated with brain ID may be rectified or at least limited with increased Tf transport of iron. In patients and animal models with ID, Tf is increased, and TfR1 expression is increased in neurons and endothelial cells [26,53], suggesting an adaptive response to increase iron acquisition via Tf.

Tf levels in the brain are also decreased in cerebral inflammation [124]. Cerebral inflammation occurs in diseases that include multiple sclerosis [125], AD [126], and depression [127]. A reduction in Tf levels may result in decreased iron delivery to neurons and relative metabolic dysfunction. 

Several cross-sectional studies did not find differences in blood transferrin levels between healthy controls and AD patients [128,129,130]. Interestingly, blood transferrin levels were positively associated with the Mini-Mental State Examination (MMSE) scores in AD patients by Fischer et al. [128] but not by Squitti and colleagues [130]. Recently, it was shown that no significant differences in the plasma Tf levels across control, mild cognitive impairment (MCI), and AD groups but higher plasma Tf levels were associated with a steeper cognitive decline in MCI and AD patients [131].

Furthermore, it was shown that disturbed brain iron metabolism is reflected in the periphery by a decrease in plasma iron and Hb [108] and a decrease in plasma iron in AD patients related to Tf desaturation was assessed, providing the potential role of Tf saturation as an AD biomarker [132].

The reasons for decreased plasma Tf saturation in AD remain unclear, though it appears indicative of a more widespread imbalance in metal homeostasis and also links with Cp activity, requiring further investigation.

## 4. Serum Biomarkers as Indicators of Iron Status

Specific self-standing indicators of iron status in the serum are difficult to fully rely upon because they span on different scales of measures and because of the presence of many confounding factors ranging from inflammation to analytic challenges. Moreover, iron status is a continuum from iron deficiency anemia (IDA) (i.e., reduced hemoglobin in red blood cells) to ID (i.e., depleted iron stores) to iron overload, and a combination of different indexes may be more useful than others depending on the situation. Available indicators include concentrations of hemoglobin, serum ferritin (s-F), soluble transferrin receptor (sTfR), zinc protoporphyrin, reticulocyte hemoglobin, serum iron, as well as total iron-binding capacity (TIBC) or transferrin saturation (TSAT) [133]. Standards serum-based indicators are generally evaluated on fully automated clinical analyzers available in most hospitals. In addition, alternative methods and new protocols for iron evaluation mainly based on analytical techniques are in continuous development. However, the standardization of methodologies (e.g., immunoassays) is complicated, and the presence of physicochemical reference methods to establish true concentrations is challenging, even if international reference materials are available [133]. Additional investigation of methods, relative analytic standardization, and interpretation of indicators are necessary for a better assessment of iron profile. Details on typical range of values of iron indicators and relative techniques to obtain these are given in Table 2.

### 4.1. Serum Ferritin

Although many indexes are available, determination of iron status by using s-F concentrations is the most commonly deployed strategy used in clinical and public health settings [134]. Ferritin is an iron storage protein and, consequently, higher intracellular iron concentrations result in increased ferritin expression, whereas ID inhibits it [135]. However, ferritin is also an acute-phase protein, and serum concentrations are increased in conditions of inflammation [136], presenting a limitation for this indicator. During liver damage, ferritin leaks from hepatocytes, and plasma concentrations rise. The ferritin measurable in the serum appears to be chiefly derived from macrophages [137] and reflects overall storage iron and ferritin concentrations in the liver and other tissues [138].

**Table 2:** Table for the standard and non-standard iron indicators. Abbreviations: CSF: cerebrospinal fluid; s-F: serum ferritin; HS: healthy subjects; M = men; W = women; Tf: transferrin; s-Tf: serum transferrin; TIBC: total iron binding capacity; UIBC: unsaturated iron binding capacity; NTBI: non-transferrin bound iron); Ft: ferritin; S: standard; NS: non-standard; HPLC: high performance liquid chromatography; MS: mass spectrometry; AAS: atomic absorption spectroscopy.

Marker	Tissue	Normal Concentration	Standard/Non-Standard	Techniques
s-F	Serum	Mean s-F concentration of adults by sex and age: 56 μg/L (W) 121 μg/L (M) [139], 120.25 ± 3.46 μmol/L (Ref. 15–200 μg/L (M) 30–370 μg/L (W)) [108]	S	Enzyme immunoassays (IA) [140]; agglutination assay: turbidimetric, nephelometric, latex photometric IA [141]. Sandwich IA using direct chemiluminometric technology [110].
Tf	Serum	204–360 mg/dL [142], 32.96 ± 0.18 μmol/L (Ref. 23–46 μmol/L) [108]	S	IA; MS analysis [143,144]. Iron-binding proteins evaluated by chromatography-inductively coupled plasma-mass spectrometry (ICP-MS) [132].
Indirect s-Tf concentration: TIBC, UIBC	Serum	TIBC: 250–400 μg/dL; 42.0–64.3 μmol/L [145]	S	Colorimetric tests [145]. Fully automated TIBC Assay [146].
Serum Iron: ferric iron (Fe^3+^) bound mainly to s-Tf		12.5–26 μmol/L (M); 10.5–23 μmol/L (W) [147]	S	Chemistry analyzers, i.e., colorimetric reaction with ferrine or ferrozine as a chromogen to form a color complex with iron.
NTBI	Serum	0.21 ± 0.10 μM [148]	NS	Ultrafiltration ICP-MS [149]; HPLC [148]; isotope dilution MS [150]; novel NTBI measuring system using nitrilotriacetate and PSAP as chromogen [151]; chelatable fluorescent beads based on flow cytometry [152]; fluorescent bead assay CP851 chelator based [153].
Total Iron	Serum	~1 mg/L	NS	AAS, ICP-MS [154,155,156].
Ft	CSF	HS: 6.4 ± 2.1 ng/mL [111] 10.7–16.4 ng/mL [157]	S	IA
Tf	CSF	0.10–0.28 µmol/L [158]; (2.3-3.6) mg/dL; ~2 mg/L [157,159]	S	Nephelometry [157]. Lectin microarray, HPLC, matrix assisted laser desorption/ionization-time of flight MS, and tandem MS [160,161].
Total Iron	CSF	0.29–1.12 µmol/L [158]; 0.12–2.00 μM [162] (26.5 ± 9.9 μg/L) in neurological control [163]	NS	Colorimetric analysis; ICP-MS [164], AAS [162,163].
Lactoferrin	Saliva	Control subjects (10.24 ± 1.96 μg/mL) [165] (7.7 ± 2.4 μg/mL) [166]	NS	MS; IA
Total Iron (indirect)	Brain	Putamen: (56 ± 11 s^−1^) Globus Pallidus: (72 ± 10 s^−1^) Caudeate Nucleus: (40 ± 6 s^−1^) Thalamus: (30 ± 6 s^−1^) (*postmortem* study) [167]	S	MRI (R2* s^−1^)

### 4.2. Serum/Plasma Tf, Serum Iron, and Tf Saturation

It is customary to measure Tf concentration indirectly from TIBC of plasma. A TIBC test measures the blood’s ability to attach itself to iron and transport it around the body, indicating the maximum amount of iron needed to saturate plasma or serum Tf. TIBC correlates well with Tf concentration, and the theoretical ratio of TIBC (in mol/L) to Tf (in g/L) is 25.1: TIBC (mol/L) = 25.1 × Tf (g/L) [168]. Theoretically, 1 mol of Tf (average molecular mass, 79 570 Da) can bind 2 mol of iron (55.8 Da) at two high-affinity binding sites for ferric iron.

Serum iron concentration measures the amount of ferric iron (Fe^3+^) mainly bound to serum Tf but does not include the divalent iron contained in serum, e.g., hemoglobin residues. It was reported that serum iron levels were significantly lower in AD patients than in healthy controls after excluded two studies [169].

Transferrin saturation (TSAT) provides additional information for the evaluation of iron transport and about the adequacy of iron supply. In blood, iron bounds with Tf provided 30% of the total transferrin is in the form of apo-transferrin [24]. TSAT indicates the percentage of binding sites of all the Tf molecules occupied by iron and is calculated as the ratio (serum iron)/Tf or (serum iron)/TIBC. If the unsaturated iron-binding capacity (UIBC) is measured, then TIBC is calculated as the sum of serum iron and UIBC [133].

It is reported that the average value of TSAT is 25% [170]. Reference ranges depend on multiple factors such as age, sex, race, and test devices. Normal values for TSAT are in the range of 25–45% (typically 30%) [171], and lower values of TSAT are reported for female (i.e., 25–27%) with respect to male (28–31%) [172]. In humans, values of TSAT < 15% indicate iron deficiency, whereas TSAT > 45% are consistent with iron overload [3]. A relatively low TSAT in conjunction with its high affinity for iron makes Tf able to efficiently buffer any alterations in plasma iron levels and to capture unshielded iron, minimizing the risk of toxicity. Above 50%, the risk of the presence of toxic NTBI rises exponentially, potentially causing organ damage [173,174].

Non-standard approaches allowed a more specific evaluation of iron-Tf binding than the indirect clinical assay, as other biological iron ligands (such as ferritin) are isolated from the iron–Tf complex, providing interesting consideration in potential AD biomarkers. In the case of AD, the changes in iron–Tf binding were masked by the poor specificity of the clinical TSAT assay [132].

Soluble transferrin receptors (sTfR) are proteins found in the blood that are cleaved from the membrane-bound Tf receptors found on nearly all cells, and the level of serum sTfR is closely related to cellular iron demands and the erythroid proliferation rate [175]. Concentrations of sTfR are inversely related to iron status; sTfR elevates in response to iron deficiency and decreases in response to iron repletion. Because TfR expression is upregulated when a cell needs more iron and because sTfR is proportional to total TfR, concentrations of sTfR are increased in plasma or serum of an iron-deficient subject [176]. Together, sTfR and s-F concentrations can cover the full range of iron status [133].

To sum up, in the condition of iron overload, serum iron and TSAT are increased and sTfR and Tf reduced, and vice versa in IDA.

### 4.3. Other Non-Standard Serum Biomarkers

The relevance of other iron status indicators (e.g., NTBI, hepcidin) proposed in the last years is under investigation [133], and laboratory methods require further improvements in terms of comparability. In particular, NTBI requires a more detailed definition of its clinically most relevant forms.

Free iron in the form of NTBI in the circulating blood becomes detectable only when Tf reaches 70% of saturation [173,177,178] and can cause significant damage to cells, even at very low concentration [173], due to its ability to catalyze the formation of ROS. NTBI is a heterogeneously speciated plasma iron and accounts for all forms of plasmatic iron bound to ligands other than Tf. Although the exact chemical nature of NTBI remains elusive, it is thought to circulate in the plasma in a form that is loosely bound to albumin or small organic acids, such as citrate [173,179]. This NTBI, iron bound to low-molecular-weight proteins or other compounds, usually comprises <1% of the plasma total iron pool and is usually not detected in most routine assays [133].

A clinically relevant level of sensitivity is yet to be achieved, although the standardization of methods to accurately quantify NTBI can be useful in order to investigate its nature and possible health effects. To date, no gold standard methods for serum NTBI quantification are established, facing technical difficulties related to the determination of heterogeneous forms of circulating NTBI and a relatively poor agreement between assays [133].

Routine clinical analysis is normally based on colorimetric tests, although other quantitative measures of total iron in serum with non-standard analytical techniques are possible, allowing advantages in precision and accuracy, simpler processing, and quicker analysis time. Their main drawbacks are that they are not commonly available in clinical laboratories and are relatively expensive, requiring regular and periodic technical maintenance together with hard pre-treatments of the blood/serum samples due to the presence of organic compounds.

Values for iron in serum were found in the order of 1 mg/L. Quantification of iron in serum of healthy, MCI, and AD subjects was performed in several studies [155,156].

## 5. Iron Markers Related to Ferroptosis

Finally, due to the emerging and relevant role of ferroptosis in neurodegeneration, it is pivotal to propose novel therapeutic approaches in AD and other neurodegenerative diseases [180]. Due to several factors associated with the ferroptotic process [181], its explicit identification in vivo is hampered by the absence of specific biomarkers. However, iron is required for the accumulation of lipid peroxides and the execution of ferroptosis. Thus, iron import, export, storage, and turnover impact ferroptosis sensitivity [93].

Three essential hallmarks define ferroptosis: the loss of lipid peroxide repair capacity by the GPX4, the availability of redox-active iron, and oxidation of polyunsaturated fatty acid (PUFA)-containing phospholipids [182].

Tf and TfR, which import iron from the extracellular environment, are required for ferroptosis [183] while silencing of the iron metabolism master regulator IREB2 decreases sensitivity to ferroptosis [88]. Furthermore, anti-TfR1 antibodies can detect ferroptosis by immunofluorescence and flow cytometry in tumor cells [184].

Interestingly, in a different pathology (amyotrophic lateral sclerosis), blood-based prognostic indicators using an array of pathological markers closely associated with ferroptosis in plasma samples were evaluated, and the identified markers of neuronal integrity, DNA, lipid oxidation, as well as iron status at baseline enabled the accurate forecasting of functional decline [185].

The development of ferroptosis-based markers is particularly timely, as iron chelation [180] and potential anti-ferroptotic therapy are currently under investigation for a range of neurodegenerative conditions, including AD.

## 6. CSF Biomarkers for Iron Status

Although it is less common to evaluate the same iron indicators for plasma/serum in CSF, it has the advantage of being in direct contact with the brain and thus potentially better reflects its iron content. One of the main limitations of CSF samples is their invasive collection technique (e.g., lumbar puncture) compared with blood sampling, and the low amount of iron requires very sensitive and reliable methods. Moreover, further investigation on the relation between serum/blood iron indicators and CSF iron indicators may be advantageous to define an iron profile for CSF.

### 6.1. CSF Ferritin

It is hypothesized that CSF ferritin can reflect global brain iron [111] and can be considered a surrogate marker of brain iron load, even if is not established as a relative biomarker [112]. However, CSF ferritin values did not show differences between cognitively normal, MCI, and AD population [111].

Interestingly, Connor and colleagues studied the potential communication of peripheral iron status to CSF, showing that iron transport correlates positively with plasma Hb concentrations but not with s-F levels [186]. The study suggested that erythropoietic demands for iron take are predominant over brain requirements, and therefore total body iron store indicators (e.g., s-F) may not be the best peripheral indicator to evaluate brain iron uptake [186].

### 6.2. CSF Transferrin

The majority of CSF iron is bound to Tf, and iron levels in this compartment are several orders of magnitude lower than in serum [187]. Additionally, it was suggested that Tf saturation in the CSF is much higher than in the periphery and that a larger proportion of NTBI circulates in the CNS [188].

CSF contains glycan isoforms of Tf; one appears to be derived from the brain and the other from the blood. The ratio of serum-type/brain-type Tf differentiates AD from idiopathic normal pressure hydrocephalus, elderly dementia caused by abnormal metabolism of CSF [160]. Immunohistochemistry using an anti-Tf antibody suggested that brain-type Tf derived from choroid plexus, a CSF-producing tissue [160]. It is hypothesized that brain-type Tf secreted from the choroid plexus would be the alternative supply of iron to neurons, being potential biomarkers for various neurological diseases [161].

Furthermore, altered glycosylation of CSF Tf molecules could be present in AD. Tf glycosylation is thus a potential biological marker for AD diagnosis, and changes in this activity may play an important role in AD pathophysiology [189].

Few studies reported values of Tf in CSF, requiring further investigation on the related biochemical status and mechanisms.

### 6.3. Non-Standard Measurements for Iron in CSF

Accurate evaluations of iron in CSF can be performed by FerroZine colorimetric analysis and related Ferrochem II analyzer methods for iron qualification in CSF or in ICP-MS [164] and AAS [162,163] for a more quantitative measure [154].

Interestingly, changes in CSF reactive iron (as redox-sensitive labile iron), crucial for the generation of free radicals, are associated with different stages of cognitive and functional impairment, but total CSF iron did not present significant alterations [162].

A recent systematic review performed by Cicero and colleagues highlighted the conflicting results in the evaluation of metal concentrations such as iron in different biological samples (i.e., blood/serum/plasma and CSF) [190]. This fact could be due to differences in the treatment of samples and in the methods used to quantify iron in biological fluids.

While NTBI is usually not detectable in the plasma of healthy individuals, it seems to be a normal constituent of brain interstitial fluid, acting as an important source of iron for several cell types in the CNS. Even under normal conditions, due to the full saturation of Tf in the CSF [191], NTBI is considered to be a physiological form of iron. As a matter of fact, iron in CSF occurring in two forms, Tf-Fe and low molecular weight NTBI (less than 30 kDa), was evaluated in rats of different ages injected intravenously with [^59^Fe^125^I]Tf [192].

It is not clear whether NTBI is generated locally or is transported from the blood across the BBB and the BCSFB, and direct evidence was reported for the transport of NTBI to the brain, also revealing a complex interplay between inflammation and brain iron homeostasis [193]. However, few studies are present, and further investigation on the quantification of NTBI and redox-active iron in CSF is essential since NTBI could be a crucial indicator, being responsible for a toxic environment contributing to neurodegeneration and neuronal death.

## 7. Iron Markers in Other Biological Fluids

Alternative non-invasive approaches involve the collection of other biological fluids, such as salivary samples. Saliva is one of the body’s mechanisms of defense due to its composition of antimicrobial proteins, and it can be used as a potential diagnostic fluid. Interestingly, a role was reported for salivary lactoferrin (LF), a glycoprotein with broad-spectrum antimicrobial activity and iron-chelating properties, preventing iron deposition [194]. LF was validated as a new single saliva biomarker, which can help the early diagnosis of MCI and AD [165]. It was assessed that decreased salivary LF levels are specific to AD, proposing that salivary LF represents one of the main defense lines against pathogens. Therefore, related low levels may worsen AD risk [166]. Low salivary LF might be an effect of immunological disturbances in AD, promoting the transfer of oral bacteria and tissue inflammatory mediators to the brain [194].

## 8. Imaging (MRI) Biomarkers for Iron Status

Brain imaging technologies are used to show that increased iron deposits in different brain regions might be markers of tissue damage in several neurological diseases [195]. MRI is the gold-standard methodology to assess and map brain iron in vivo.

Hydrogen nuclei (e.g., protons) abundant in fat and water are the main components of the human body and possesses a spin, generating a small magnetic moment interacting with properly designed external fields.

In particular, MRI acquisition employs a large magnet producing a strong magnetic field (B_0_) much larger than the geomagnetic field. Within a body positioned inside the scanner, B_0_ aligns the protons’ spins along its direction. At equilibrium, the net magnetization corresponds to the longitudinal magnetization along the sagittal axis of the body and is parallel to B_0_. Depending on the selected “sequence”, the net magnetization vector is suddenly changed by exposing the spins to radiofrequency pulses modifying their orientation (e.g., overturning the vector onto the xy plane). Once the pulses are removed, the protons tend to return to their equilibrium position according to tissue-specific exponential relaxations. Two time constants are normally considered: the longitudinal, or spin-lattice, relaxation time (T_1_), and the transverse, or spin-spin, relaxation time (T_2_). In real settings the T_2_ decay actually results from two sources: molecular (spin-spin) interactions (pure T_2_) and variations in B_0_ that may lead to inhomogeneities in T_2_ effect. The time constant that characterizes these two processes together is called T_2_*.

By applying different sequences of radiofrequency pulses and collecting the resulting signal, images with different contrasts can be reconstructed. In particular, gradient-echo T_2_*-weighted images are usually generated by multiple short echo times, small flip angles, and variable repetition times.

Starting from these standard MRI techniques, combinations of sequences and images reconstruction strategies may produce more specific mappings.

Quantification of iron levels can be carried out using several techniques, such as relaxation time mapping [167,196], quantitative susceptibility mapping (QSM) [197], magnetic field correlation [198], and direct saturation imaging [199]. Many different MRI acquisition schemes are due to the fact that the presence of iron in the brain has different effects: (i) changes in relaxation characteristics of tissue water in presence of ferritin; (ii) changes in tissue susceptibility changes induced by iron presence; and (iii) microscopic field gradients in its surroundings. The nature and the entity of these changes depend on the different magnetic properties of iron compounds and may affect image contrast in terms of quantitative relaxation parameters and of the phase of the complex MRI signal [200].

**Figure 5:**
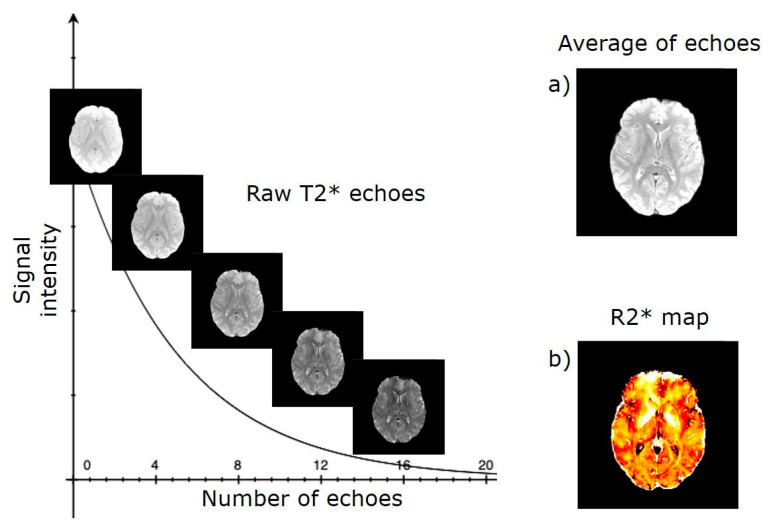
MRI processing workflow of T_2_* to map brain iron. (**a**) Each echo is aligned on the average image for motion correction. (**b**) Realigned echoes are fitted through a voxel-by-voxel nonlinear least squares model to obtain a mono-exponential signal decay curve $S=S_{0}e^{-\frac{t}{T_{2}*}}$ and finally produce a transverse relaxation rate map (R_2_*).

Since the earliest MRI experiments, it was observed that iron mainly accumulates in the grey matter of the brain, which appears hypointense on T2-weighted MRI. Subsequently, several studies reported associations between age and the transverse relaxation rates R_2_ = 1/T_2_ and R_2_* = 1/T_2_*, which are commonly used as surrogate markers for iron concentration in brain tissue. In particular, Langkammer and colleagues [167] investigated the relationship between these measurements and chemically determined iron concentrations in seven human brains post mortem. They found that the basal ganglia (pallidum, putamen, caudate nucleus) and the thalamus were the regions with the highest iron concentration and that the R_2_* rates showed the strongest linear correlation with chemical measurements throughout the brain (r2 = 0.90, *p* < 0.001). These results support the fact that R_2_* is more sensitive than R_2_ to variations in brain iron concentrations and might be considered the preferred parameter for the in vivo assessment of brain iron concentration. Figure 5 shows the typical processing workflow of T_2_*-weighted data.

In healthy subjects, a recent study [201] identified R_2_* measurements as the best age predictors in specific regions of the brain, such as the putamen and the globus pallidum, confirming the results of the abovementioned post-mortem study. Moreover, another recent systematic review [202] suggested that T_2_* and R_2_* sequences might also be useful to increase the accuracy of automated segmentation of subcortical brain structures, especially when the T_1_-weighted contrast is reduced (e.g., due to age-related effects). About neurodegenerative diseases, Langkammer and colleagues investigated the currently available MR methods for quantitative iron mapping in the brain of patients with AD [40], suggesting R_2_* mapping as the best-validated technique for iron detection. In particular, these patients show increased iron levels not only in the hippocampus and the temporal cortex, damage of which is a well-known hallmark of the disease, but also in the pulvinar thalamus (connected to the visual cortex) and in the putamen and the red nucleus (both involved in motor control).

The role of iron accumulation as a potential imaging marker in patients with MCI or AD was also suggested using susceptibility-weight imaging, since motor cortex hypointensity on this sequence was more frequently found in patients with cognitive impairment than in age-matched controls and was also positively associated with age [203].

Among novel methods for iron detection in the brain, QSM was used for in vivo longitudinal monitoring of Aβ accumulation and iron deposition in a transgenic mouse model of AD [204]. Increased iron concentrations correlate with Aβ aggregation in areas initially affected in AD and offer an opportunity for MRI-based diagnosis. MRI scans of post-mortem human brains and a mouse model of AD showed decreases in hippocampal T_2_* MRI, which is sensitive to the magnetic properties of iron [205] or its spatial variance, attributed in part to iron in Aβ plaques [206]. Although MRI resolution is not sufficient to detect individual plaques, T_2_* abnormalities that result from plaque aggregates might be detected with MRI. When clearly distinguished from potential confounders originating from heme iron, changes in hippocampal T_2_*-weighted MRI might be a valuable assessment of morphological changes and a potential biomarker for the early stages of AD [207].

Other findings of evidence for iron overload in several specific brain regions were reviewed by Tao et al. [154].

## 9. Conclusions

The aim of this review was to summarize the complexity of iron metabolism, especially related to the blood–brain exchange, and the dysregulation of several iron-related parameters involved in neurodegeneration. We proposed an up-to-date screenshot of the parameters presently used as indicators (routinely or not routinely assessed in clinical practice) of iron status in biological fluids, such as serum and CSF, in order to unravel potential iron biomarkers and their correlations in neurodegenerative diseases. Finally, we also focused on the-state-of-art of current MRI methods to evaluate the quantification of iron in the brain.

In conclusion, the evidence for the role of ferroptosis in the pathophysiology of neurodegenerative diseases is emerging, and iron metabolism is strictly involved. Exploring the relation between standard iron indicators, e.g., Tf, and novel indicators such as LF and the quantification of redox-active iron (e.g., NTBI) could be useful to reveal the cascade of events in neurodegeneration processes. Moreover, they could be integrated with other markers possibly related to ferroptosis to foster complex pathological patterns.

In particular, new potential sets of biomarkers for iron status in biological fluids together with an improvement and standardization of reliable analytical methods to detect them (e.g., speciation of iron to assess ionic and binding state) are essential to quantitively describe the processes contributing to neurodegeneration, including ferroptosis.

Finally, we believe that an accurate evaluation of the associations between iron indicators in blood and cerebral space (CSF and brain regions) by means of data-driven investigations based on the machine learning techniques or similar [163,208] could help to outline the peripheral indicators most suitable for a brain iron profile and potential alterations of iron trafficking between blood and brain.

## Figures and Tables

**Table 1:** Table for the description of iron-related proteins involved in uptake, regulation, transport, and storage of iron from blood to brain. Abbreviations: BBB —blood–brain barrier, BCSFB—blood-cerebrospinal fluid barrier, CSF—cerebrospinal fluid, Cp—ceruloplasmin, DcytB—duodenal cytochrome B, DMT1—divalent metal transporter-1, FPN1—ferroportin-1, Ft—ferritin, HEPH—hephaestin, LIP—labile iron pool, Tf—transferrin, TfR—transferrin receptor.

Body Compartment/Structure	Iron Uptake	Iron Transport/Regulation	Iron Storage
Intestinal Lumen-Enterocytes	DMT1	DcytB FPN1 HEPH Cp	Ft
Blood	Release from enterocytes (FPN1)	Tf	Ft
		Apo-Tf Hepcidin	
BBB-BCSFB		DcytB	
	TfR DMT1	DMT1	Ft
		FPN1	
		HEPH, Cp	
Brain-CSF	Entry from brain barriers TfR DMT1	Tf, Apo-Tf FPN1, DMT1 Cp	Ft

## Data Availability

Not applicable.

## Abbreviations

AD	Alzheimer’s disease
APOE	Apolipoprotein E
APP	Amyloid precursor protein
Aβ	Amyloid-beta
ATP	Adenosine triphosphate
MCI	Mild cognitive impairment
MRI	Magnetic resonance imaging
DMT1	Divalent metal transporter-1
Fpn	Ferroportin
Ft	Ferritin
Ft-L	Ferritin light chain
Ft-H	Ferritin heavy chain
Tf	Transferrin
LIP	Labile iron pool
HEPH	Hephaestin
BBB	Blood–brain barrier
BCSFB	Blood–cerebrospinal fluid barrier
BMVECs	Brain microvascular endothelial cells
CSF	Cerebrospinal fluid
Cp	Ceruloplasmin
DcytB	Duodenal cytochrome B
NTBI	Non-transferrin bound iron
NADH	Nicotinamide adenine dinucleotide
ROS	Reactive oxygen species
GSH	Intracellular glutathione
GPX4	Glutathione peroxidase 4
ID	Iron deficiency
IDA	Iron deficiency anemia
TIBC	Total iron binding capacity
UIBC	Unsaturated iron binding capacity
TSAT	Transferrin saturation
sTfR	Soluble transferrin receptor
s-F	Serum Ferritin
LF	Lactoferrin
IA	Immunoassay
HPLC	High performance liquid chromatography
MS	Mass spectrometry
AAS	Atomic absorption spectroscopy
QSM	Quantitative susceptibility mapping
